# “Procedural sedation and analgesia in Italian pediatric emergency departments: a subgroup analysis in italian hospitals"

**DOI:** 10.1186/s13052-023-01426-7

**Published:** 2023-02-15

**Authors:** Martina Bevacqua, Idanna Sforzi, Silvia Bressan, Egidio Barbi, Cyril Sahyoun

**Affiliations:** 1 University of Trieste, Piazzale Europa 1, 34127 Trieste, TS Italy; 2 Pediatric Emergency Department and Trauma Center, Meyer Children’s Hospital, Viale Pieraccini 24, 50139 Florence, Italy; 3 Department of Women’s and Children’s Health, University of Padova, Via VIII Febbraio, 2, 35122 Padua, PD Italy; 4 Institute for Maternal, Child Health - IRCCS “Burlo Garofolo”, Via Dell’Istria 65, 34137 Trieste, Italy; 5 Division of Pediatric Emergency Medicine, Children’s Hospital of Geneva, Geneva University Hospitals, Rue Gabrielle-Perret-Gentil, 4, 1205 Geneva, Switzerland

**Keywords:** Sedation, Analgesia, Italy, Emergency, Gaps

## Abstract

To date, pain and anxiety are the most common symptoms reported by children who refer to pediatric emergency department. Despite it is well known that the undertreatment of this condition has some negative consequences in a short term and long term of time, gaps in the management of pain in this setting still persist. This subgroup analysis aims to describe the current state of art of pediatric sedation and analgesia in Italian emergency departments and to identify existing gaps to solve. This is a subgroup analysis of a cross-sectional European survey of pediatric emergency departments sedation and analgesia practice undertaken between November 2019 and March 2020. The survey proposed a case vignette and questions addressing several domains, like the management of pain, availability of medications, protocols and safety aspects, staff training and availability of human resources around procedural sedation and analgesia. Italian sites responding to the survey were identified and their data were isolated and checked for completeness. Eighteen Italian sites participated to the study, the 66% of which was represented University Hospitals and/or Tertiary Care Centers. The most concerning results were an inadequate sedation to 27% of patients, lack of availability of certain medications like nitrous oxide, the lack of use of intranasal fentanyl and topical anesthetics at the triage, the rare use of safety protocols and preprocedural checklists, lack of staff training and lack of space. Furthermore, the unavailability of Child Life Specialists and hypnosis emerged. Despite procedural sedation and analgesia in Italian pediatric emergency departments is progressively more used than previously, several aspects still require an implementation. Our subgroup analysis could be a starter point for further studies and to improve and make the current Italian recommendations more homogeneous.

## Introduction

Treatment of pain and anxiety in children during medical procedures is of utmost importance, in order to provide children and their families the most comfortable and the least traumatic experience possible [1]. Despite that, gaps continue to exist in the management of children’s wellbeing during these procedures [2, 3]. An increasing number of Italian studies around Procedural Sedation and Analgesia (PSA) in children attests to the growing national importance of this field [4, 5]. However, to the best of our knowledge and to date, no studies have surveyed the state of PSA in Italian pediatric Emergency Departments (EDs). A recent European survey describing PSA practice in 19 European countries showed that although procedural sedation and analgesia are widely used in pediatric EDs, many barriers to its implementation still exist, such as the lack of availability of some medications, lack of a standardized approach, as well as staff shortage and lack of space, and external control of sedation agents used in the EDs [6].

This sub-analysis aims to describe the current status of pediatric PSA in Italian EDs, in an effort to identify existing gaps and to propose strategies to reduce them.

## Materials and methods

### Study design, setting, procedures

This study is a subgroup analysis of a cross-sectional European survey of pediatric ED PSA practice undertaken between November 2019 and March 2020 [6]. Italian sites responding to the survey were identified and their data were isolated and checked for completeness.

In the original study, in each participating country, lead research coordinators were identified based on their knowledge and experience in the field of pediatric emergency medicine. A quota sampling method was used to determine the number of sites that needed to be enrolled for each country, based on the country’s population. For countries with more than 20 million inhabitants, such as Italy, participation of at least 10 sites was targeted. Site enrollment was achieved through contacting the clinical chief or the physician in charge of PSA in the pediatric ED of each site, through emails or direct phone calls.

### Survey content

The survey started with a case vignette, then included questions addressing several domains:

- 1. Management of a theoretical patient requiring PSA.
- 2. Medication availability and frequency of use.
- 3. Characteristics of staff performing PSA and their training.
- 4. Protocols and safety aspects.
- 5. Nursing-directed triage protocols, topical anesthetics, and minor trauma care.
- 6. Human resources around PSA.
- 7. Barriers to implementation of PSA.
- 8. Staff satisfaction with their site’s PSA efforts.

### Statistical analysis

For the results, frequencies and percentages were used for categorical variables.

Given an expected disparity in the number of patients per site and seen per year, coherently with the original study, we reported the results as a proportion of the total number of children seen per year for domains involving patient-centered data (management of a theoretical patient, medications availability, characteristics of staff performing PSA) and presented them as percentages. For the same reason, domains involving site-centered data (frequency of use of sedation medications, availability of protocols) were reported as a proportion of the total number of sites. Percentages were rounded to the nearest integer.

## Results

### Respondents

**Fig. 1:**
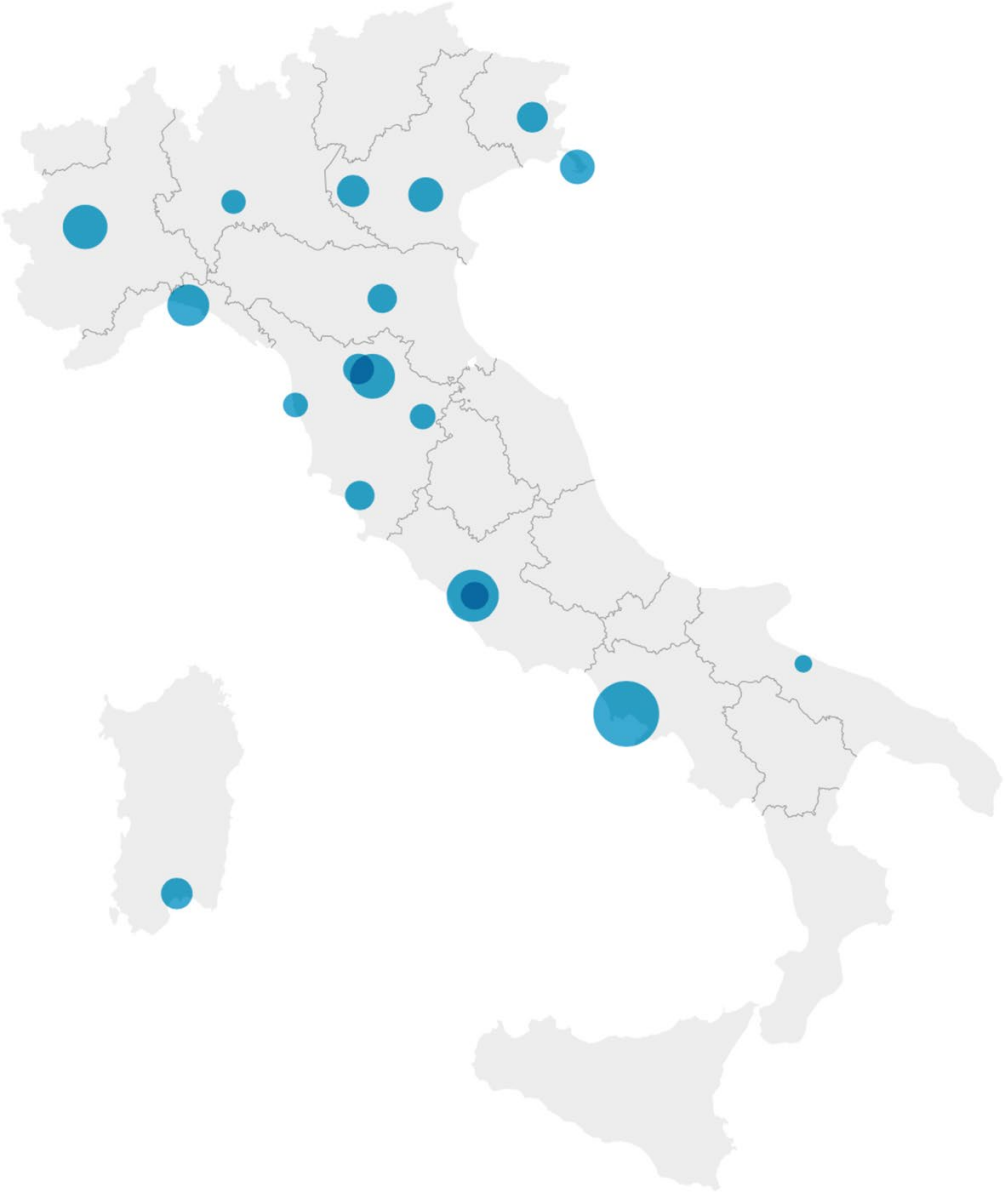
Geographic distribution of the survey participants in Italy. Each dot represents one emergency site. The diameter of each circle represents the relative number of children seen in the emergency department per year (larger dot = higher number of children)

Eighteen sites participated in Italy, representing 10 Italian regions, with a response rate of 100% according to the pre-defined target of enrolling at least 10 sites in countries with more than 20 million inhabitants, as detailed in methods above. In 2019, the mean number of children seen per site, per year, was 27,931 (95^th^ CI 17,712 – 38,150) representing a total of 502,764 patients (Fig. 1). Sixty-six percent of the surveyed sites were University Hospitals and/or Tertiary Care Centers. All centers took care of trauma patients.

### Survey Responses

#### Management of a theoretical patient requiring PSA (as a proportion of children)

**Fig. 2:**
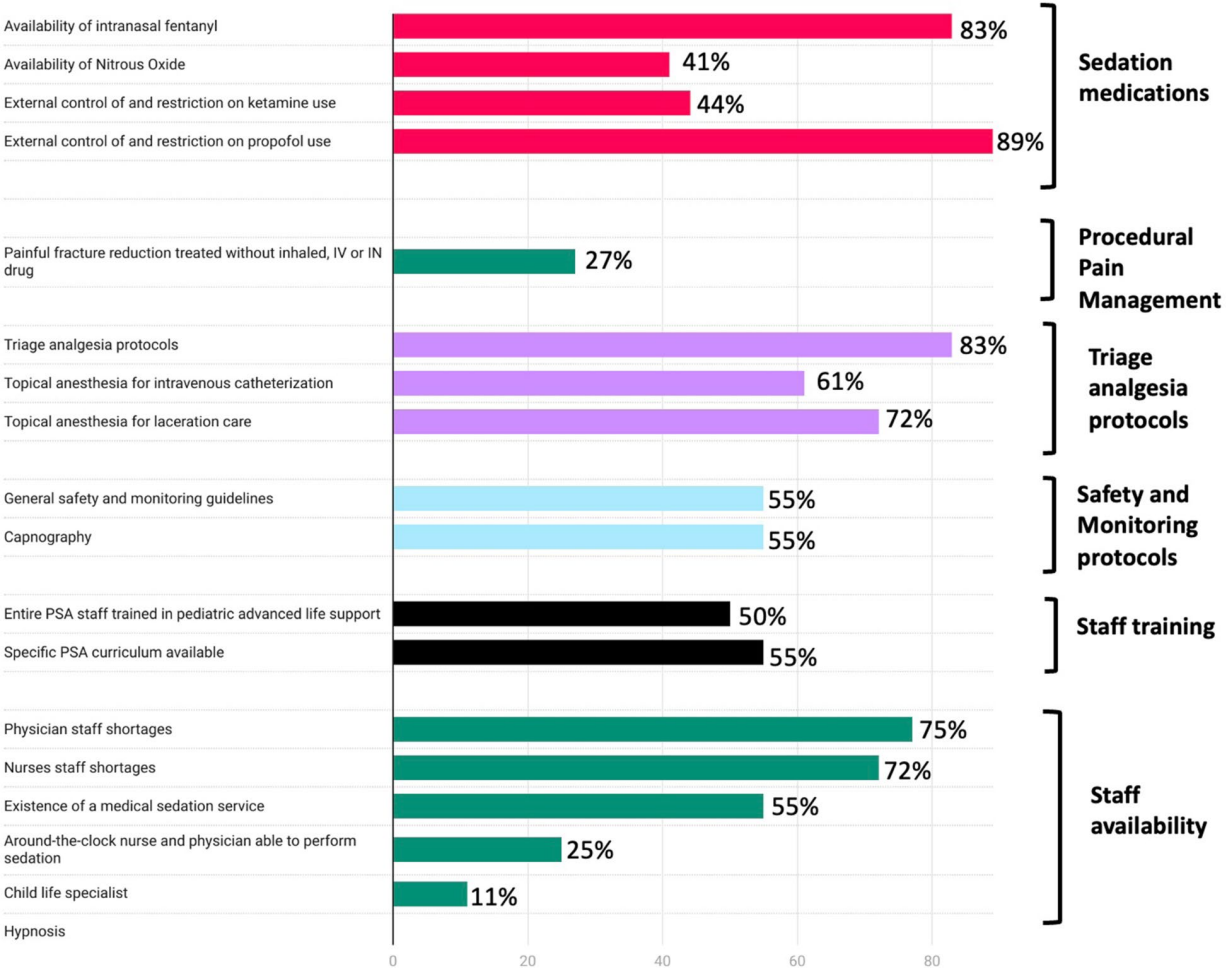
Prevalence of areas for improvement, in selected domains. *PSA: procedural sedation analgesia; IV: intravenous; IN: intranasal*

A 4-year-old patient with a displaced forearm fracture requiring closed reduction and casting would be treated as follows: intravenous (IV) sedation in the ED in 27% of centers, under general anesthesia in 13%, with nitrous oxide (NO) with or without a hematoma block and with or without intranasal (IN) fentanyl in 19%, and with intramuscular sedation in 2% of centers (Fig. 2). The patient would be treated with analgesics and transferred to a referral center in 12% of participating centers. Children were treated without inhaled, IV, or IN medications in 27% of the cases.

#### Sedation medication availability (as a proportion of children represented) and frequency of use (as a proportion of sites)

The following medications were available for pediatric use: midazolam (intravenous: 100%, oral: 54%, intranasal: 86%), ketamine (96%), and propofol (52%). Other intranasal medications available for pediatric use included fentanyl (83%), and dexmedetomidine (41%). Nitrous oxide was available to 41% of the children (Table 1).

**Table 1:** Availability of selected medications and routes in Italian emergency departments

	*As a proportion of sites surveyed*	*As a proportion of children represented*
**Ketamine**		
- IV	17 (95%)	483,764 (96%)
- IN	6 (33%)	179,764 (36%)
- At least one route	17 (95%)	483,764 (96%)
**Midazolam**		
- IV	18 (100%)	502,764 (100%)
- IN	16 (89%)	431,764 (86%)
- PO	13 (72%)	272,264 (54%)
- At least one route	18 (100%)	502,764 (100%)
**Nitrous oxide**	9 (50%)	207,690 (59%)
**Propofol**	11 (61%)	270,838 (54%)
**Fentanyl (at least one route)**		
- IV	14 (78%)	361,264 (72%)
- IN	16 (89%)	417,764 (83%)
**Dexmedetomidine IN**	6 (33%)	205,500 (53%)
**Chloral hydrate**	Not available	Not available
**Topical anesthetics**		
- For laceration care	13 (72%)	382,264(76%)
- For intravenous catheterization	11 (61%)	356,000 (71%)
**Tissue adhesive**	16 (89%)	472,764 (94%)

**Legend:** IV intravenous, IN intranasal, PO per Os.

Where available, intravenous sedation was used less than once a week in 31% (5/16), up to 2–6 times a week in 63% (10/16) and more than 5 times a day in 6% (1/16).

Nitrous oxide was used less than once a week in 33% (3/9), once to 4 times a week in 56% (5/9), and 2 to 4 times a day or more in 11% (1/9). Equimolar 50% nitrous oxide/50% oxygen was the only used mixture, with none of the sites sanctioned to use 70%/30%.

#### Characteristics of staff performing PSA (as a proportion of children and sites) and training (as a proportion of sites)

Children were sedated by general pediatricians in 78% (14/18 sites), anesthesiologists in 72% (13/18 sites), Pediatric Emergency Medicine (PEM) physicians in 44% of cases (8/18 sites), general emergency physicians (treating children and adults) in 33% (6/18 sites), and pediatric intensivists in 22% (4/18).

Specific PSA courses, in addition to pediatric advanced life support (PALS) courses, were required for the staff administering PSA in 55% (10/18) of the sites, while a specific number of supervised PSA cases was required in 44% (8/18) before performing PSA independently. In 50% (9/18) of the sites, the entire physician staff performing PSA were Pediatric Advanced Life Support (PALS) certified, in 16%, less than half and less than a quarter were certified.

Trainees were allowed to administer PSA during their training in 39% (7/18) of the sites. Of these, in 57% (4/7) of sites this was allowed for senior residents only (in their 5^th^ year of pediatric training).

#### Protocols and safety aspects (as a proportion of sites)

General safety and monitoring guidelines (defined as local protocols, written internally by the site, aimed at standardizing the practice around procedural sedation and analgesia, and containing detailed indications and contraindications for sedation, staff required to be present in the room during the procedure, monitoring equipment requirement, etc.) were available in 55% (10/18), and pre-procedural checklists (a specific checklist of material, adjunct medications, and information that needed to be prepared or obtained in preparation for the sedation) in 49% (8/18). Capnography (via nasal-oral cannula) was available in 55% (10/18) of the sites, and occasionally used in 60% (6/10). During PSA with IV Ketamine, in sites that used the medication, physicians administered the medication in 59% (9/17), nurses in presence of a physician in 29% (5/17), and either one in 12% (2/17).

#### Nurse-directed triage analgesia protocols (as a proportion of sites), topical anesthetics and minor trauma care (as a proportion of children)

Nurse-directed triage analgesia protocols (a protocol or standing order allowing nurses to give analgesics at triage without prior medical prescription) were in place in 83% (15/18) of sites. Of those, the protocol included paracetamol in 100% (15/15), ibuprofen or similar non-steroidal anti-inflammatory drug in 80% (12/15), and an oral opiate or IN fentanyl in none. Topical anesthesia for lacerations (lidocaine, epinephrine/adrenaline, tetracaine or similar) was available to 76% of the children, while for intravenous catheterization (Eutectic Mixture of Local Anesthetics, EMLA or similar), it was available to 71%. Tissue adhesive for laceration repair (such as Dermabond, SurgiSeal) was available to 94% of the children.

#### Human resources around PSA (as a proportion of sites)

The availability, at any time of the day, of a physician able to perform PSA was 39% (7/18) for single coverage (one individual present at any given time), also 56% (10/18) for double coverage (two individuals present at any given time) and 6% (1/18) for triple coverage. Nurse availability was 33% (6/18) for single coverage, 39% (7/18) for double coverage. No nurses were available in 11% of sites (2/18). ED physicians sedated outside the ED in 28% (5/18) of the sites. A formal medical sedation service for elective sedations (a team sedating patients from different services of the hospital, such as ward, radiology or other interventional services) was available in 55% (10/18); staffed by anesthesiologists in 80% (8/10), general pediatricians in 40% (4/10), pediatric intensive care medicine physicians and pediatric emergency medicine physicians in 10% (1/10) of the sites.

Child life specialists (CLS) was available in only 11% (2/18) of the sites. Hypnosis was available in none of the sites.

#### Barriers to implementation of PSA (as a proportion of sites)

Nurses and physicians staff shortages were reported in 72% and 77% (13/18 and 14/18) of sites respectively, and lack of physical space in 83% (15/18) of sites. Anesthesiologists controlled or restricted ketamine and/or propofol use (defined as the ED not being free to create a protocol and use the medication without direct supervision or official approval) in 44% (8/18) and 89% (16/18) of the sites, respectively. All respondents (18/18) agreed that ketamine was a useful agent for PSA in the ED.

#### Staff satisfaction around PSA (as a proportion of sites)

Fifty percent (9/18) of respondents reported to be satisfied with their site’s management of pain and anxiety in children.

## Discussion

In this subgroup analysis of data extracted from a survey looking at the current pediatric PSA practice in Europe, we focused on the current practice of PSA in Italian pediatric EDs.

Our results show that a large proportion of children who require PSA do not get offered adequate sedation, that general safety and monitoring guidelines and preprocedural checklists are not always systematically available, and that additional barriers to implementation included staff shortage, lack of medications, absence of child life specialist services and hypnosis and lack of space. Furthermore, a point of concern emerged by our analysis is the restriction to the anesthesiologist of the administration of certain medications, such as ketamine, despite it has been proved to be the safest single agent for PSA, indicated even for urgent procedures, when pre-procedural fasting is not possible to perform [7].

A low level of satisfaction with their site’s management of pain and anxiety was also reported by respondents, which may be harnessed as a driving force for improvement.

In the following discussion we analyze the identified gaps and propose a problem-solving strategy of the major challenges emerged by this subgroup analysis.

### Inadequate sedation to 27% of patients

As mentioned above, the most disconcerting result emerging from our analysis is that an adequate sedation plan is not used in 27% of patients who require it, in an era where pain assessment and management has been finally recognized as priority for the best patient quality of care, and backed by multiple international bodies, including the Joint Commission [8].

It is a known fact that a proportion of children who access the pediatric ED live a painful and unpleasant experience [1, 9, 10], not only related to the reason of the medical consultation, but also due to medical procedures performed during the clinical evaluation and treatment. At the same time, pain is one of the most frequent reasons of referral to pediatric EDs, especially in younger children and in those with special needs, a category in which undertreatment of pain (the so-called “*oligoanalgesia*”) is very frequent [1, 11, 12]. Given that *oligoanalgesia* is related to long-terms negative behavioral and psychological consequences, [1, 13, 14] and that the management of pain and anxiety could help the entire medical team in the evaluation and treatment of a child, we identify this gap as a major source of potential improvement, in a continued effort to make pediatric EDs pain-free or at the very least free of iatrogenic traumatic experiences.

### Lack of availability of certain important medications

Our analysis also points to the low availability of certain types of medications such as nitrous oxide, and the non-systematic availability of topical anesthetics.

### Lack of use of Nitrous oxide

NO is a safe and prompt-to-use gas, shown to be useful in performing many painful procedures as fracture reductions, sutures or placing an intravenous catheter, alone or in combination with other medications such as intranasal fentanyl or intranasal dexmedetomidine, especially in an emergency setting [15]. Despite that, NO was reported to be available only to less than a half of the children represented by this study. Since many studies showed the safety and efficacy of this medication, as much as a high degree of satisfaction of the patient and the parents, [16, 17] we firmly encourage an implementation of its use in Italian pediatric EDs.

### Oral opioids, intranasal fentanyl and topical anesthetics: a useful tool since the triage

Intranasal medications as fentanyl and midazolam are available for almost the totality of the children represented in our study, confirming the growing importance and use of these agents in performing PSA in pediatric EDs, likely thanks to their ease of administration. However, another major issue concerns the absence of these medications in nurse-directed triage analgesia protocols. Furthermore, topical anesthetics are unexpectedly reported to be rarely used in Italian pediatric EDs.

The administration of an analgesic medication at the time of triage has been shown to improve the management of pain by reducing the time to reach adequate analgesia and by making the medical evaluation easier. Nurse-directed triage analgesia protocols were available in almost the totality of the Italian sites participating in this study, but they only included topical anesthetics, oral ibuprofen or similar non-steroidal anti-inflammatory drug and paracetamol. These are medications with a relatively long time of onset and adequate mainly for mild pain. Intranasal fentanyl, a very effective, safe and easy to administer medication with an onset of action of only 3–5 min, despite being available to almost the totality of children in our study, is not included in nurse-directed triage protocols [18–21]. To quickly ease severe pain, we would strongly encourage the introduction of oral opioids as well as intranasal fentanyl to nurse-directed triage analgesia protocols backed by a standing medical prescription, in conjunction with safety guidelines around their use (indications, contraindications, monitoring, etc.). These would help decrease the time to analgesia for painful conditions (such as fractures, vaso-occlusive crisis, severe abdominal pain) already at the first nurse evaluation, particularly when the ED is busy and a bed not immediately available.

### Off label use of medications

Another barrier in access to and implementation of medications is that many drugs used for pain and anxiety control are off label for the pediatric age, or have limited applications [22]. Despite the recent increase in publications around PSA, specific studies in the pediatric age remain scarce. Consequently, the off-label use of such medications requires informed consent, and their use are under the responsibility of the practicing physician. Depending on the training and experience of that physician in pediatrics, and depending on the site of practice, such use may create a medico-legal concern, as should, inarguably, inadequate pain and anxiety relief during procedures. We believe that the creation of PSA national guidelines for pediatricians but also for staff intervening in emergency situations but without pediatric expertise, would help palliate such concerns.

### Rare use of safety protocols and preprocedural checklists, lack of staff training and lack of space

PSA, in our study, was performed mainly by general pediatricians and anesthesiologists. Furthermore, several PSA medications (ketamine and propofol, particularly) are restricted for use, in the ED, without direct supervision or official approval by anesthesiology colleagues. In Italy, this heterogenous practice is probably the consequence of the fact that pediatric emergency medicine is not yet formally recognized as a board-certified subspecialty at a national level, while it is recognized as such in foreign settings. Then, PSA in the Italian pediatric reality is still a developing skill, and that in order to gain more independence, efforts should concentrate on homogeneous training of the pediatric staff in PSA, on the drafting and the systematic use of national safety guidelines, as well as on the universal training of pediatricians administering PSA in a PALS course. *A latere*, the improvement of the equipment in the EDs, such as capnography monitoring, may furthermore increase the safety of PSA, during ketamine and propofol sedations, for example.

Furthermore, the use of general safety and monitoring guidelines and checklists was reported in around half of the responding sites only, making the lack of a standardized approach an issue of concern, which could expose patients to possible adverse events related to patient or sedation characteristics. For this reason, the development and implementation of local, but also standardized national guidelines around PSA should be fostered.

### Unavailability of child life specialists and hypnosis

Even if our data showed that the availability of Child Life Specialists among Italian pediatric EDs was the same as the average availability in Europe, the numbers remain small (11 vs 13%). Hypnosis, on the other hand, is reported to be completely unavailable among the respondent Italian sites. As described for the other European sites, this could be due to cultural beliefs, prioritization of resources and low experience with this field, particularly for the pediatric age.

Since children have more hypnotic ability than adults (measured by the Children’s Hypnotic Susceptibility Scale and the Stanford Hypnotic Scale for Children), several studies proposed hypnosis as a tool for procedural pain and chronic pain management in children, reporting a significative reduction in pain and anxiety and improving the patient experience [23–28].

For this reason, we recommend training in nonpharmacological techniques such as hypnosis, and increasing knowledge of the added value of child life specialists and similar professionals.

The aspects above, taken together, may explain the low satisfaction of the respondents with their site’s management of pain and anxiety in children.

## Limitations

Our study has several limitations. First, this is a retrospective subgroup analysis of self-reported practice, which makes possible some human errors in reporting and collecting the data. Second, despite efforts to avoid it, data collected by a survey methodology could be subject to sampling bias (the person responding to the survey may not be the most qualified to do so) and non-respondent bias. Our subgroup analysis includes 18 Italian sites, but in some case the same city (e.g. Rome) had more than one ED represented, while in contrast, some regions did not participate at all (e.g. no data available for Abruzzo, Marche, Molise, Basilicata, Sicily and Calabria), making the sample not completely representative of the entire country.

## Conclusions

In conclusion, even if pediatric PSA is becoming more frequently used in Italian pediatric EDs, several aspects require significant improvement, including increasing access to sedation and analgesia medications, training of staff, and expansion of nurse-directed triage protocols. Barriers of implementation noted in our subgroup analysis were lack of training, staff shortages and lack of spaces. Most importantly, the high proportion of children not having an adequate sedation plan for a painful procedure suggests the need for in-depth work by the Italian pediatric emergency medicine community.

Our subgroup analysis could be a starter point for further studies and to implement current Italian recommendations [29] at institution levels, leading to the creation of national PSA guidelines, in order to guarantee an evidenced based, effective approach to pain and anxiety for all children seeking emergency care in Italy.

## Data Availability

All data generated or analyzed during this study are included in this published article: Pediatric procedural sedation and analgesia in the emergency department: surveying the current European practice, Eur J Pediatr. 2021 Jun;180(6):1799–1813. https://doi.org/10.1007/s00431-021-03930-6.

## Abbreviations

PSA: Procedural Sedation and Analgesia
EDs: Emergency Departments
ED: Emergency Department
NO: Nitrous Oxide
PEM: Pediatric Emergency Medicine
IN: Intranasal
PALS: Pediatric Advanced Life Support
CLS: Child Life Specialists
